# Probiotic Potential in Irritable Bowel Syndrome and Inflammatory Bowel Disease: A Comprehensive Systematic Review

**DOI:** 10.7759/cureus.72089

**Published:** 2024-10-22

**Authors:** Anura Manandhar, Ghadeer Sabir, Hala A Abdelhady, Adoum Oumar Abakar, Ravindra Reddy Gangavarapu, Sayed A Mahmud, Iana Malasevskaia

**Affiliations:** 1 Internal Medicine, California Institute of Behavioral Neurosciences & Psychology, Fairfield, USA; 2 Internal Medicine and Clinical Research, California Institute of Behavioral Neurosciences & Psychology, Fairfield, USA; 3 Medical Research, California Institute of Behavioral Neurosciences & Psychology, Fairfield, USA; 4 Medicine, European University, Tbilisi, GEO; 5 Obstetrics and Gynecology, Private Clinic "Yana Alexandr", Sana'a, YEM; 6 Research and Development, California Institute of Behavioral Neurosciences & Psychology, Fairfield, USA

**Keywords:** efficacy, ibs, inflammatory bowel disease-crohn's diseases, inflammatory bowel disease-ulcerative colitis, irritable bowel syndrome, probiotics

## Abstract

Irritable bowel syndrome (IBS) and inflammatory bowel disease (IBD) are prevalent gastrointestinal disorders with significant global prevalence. Conventional treatments often have adverse effects, prompting interest in probiotics as alternative therapies. This systematic review assesses the efficacy of probiotics in managing symptoms and improving outcomes in adult patients with IBS and IBD. A comprehensive search was conducted across databases such as PubMed, Cochrane Library, and Google Scholar and registers ClinicalTrials.gov and International Standard Randomized Controlled Trial Number (ISRCTN). Using targeted keywords, studies on probiotic efficacy in adult IBS and IBD patients were identified. Data screening, extraction, and quality assessment using the Cochrane Risk of Bias 2 (RoB 2) tool for evaluating randomized controlled trials (RCTs) and Newcastle-Ottawa Scale (NOS) for cohort studies were rigorously performed following Preferred Reporting Items for Systematic Reviews and Meta-Analyses (PRISMA) 2020 guidelines. From the initial 22,037 references, 18 randomized control trials and two observational studies encompassing 2,675 adults, aged 18-76 years, were deemed eligible. The efficacy of probiotics for IBS and IBD is variable. While some IBS trials show symptom improvement, the results are inconsistent, likely due to the diversity of probiotic strains and patient populations studied. In contrast, probiotics demonstrate more consistent benefits for ulcerative colitis (UC) in IBD, particularly with specific formulations like the De Simone combination. However, probiotics' effects on Crohn's disease (CD) remain less clear, highlighting the need for further research to optimize probiotic regimens and understand their differential effects across the spectrum of IBS and IBD.

## Introduction and background

An estimated 7% to 18% of people worldwide suffer from irritable bowel syndrome (IBS), a complicated and multivariate functional gastrointestinal condition [1]. Recurrent stomach pain or discomfort linked to altered bowel habits-constipation, diarrhea, or both-is what defines IBS [1]. IBS can be divided into four subtypes based on the main bowel pattern: mixed bowel habits (IBS-M), constipation-predominant (IBS-C), diarrhea-predominant (IBS-D), and unclassified (IBS-U) [1].

Inflammatory bowel disease (IBD) encompasses two primary chronic, recurrent inflammatory disorders of the gastrointestinal tract-Crohn's disease (CD) and ulcerative colitis (UC) [2]. These two conditions represent the predominant manifestations of IBD [2]. IBD is a global health concern, with developed and industrialized regions-especially Europe and North America-observing the maximum occurrence of the disease [2]. An abnormal immune response and chronic intestinal inflammation are the results of an intricate interplay between genetic, environmental, and immunological variables in the pathophysiology of IBD [3].

Conventional pharmacological treatments for IBS and IBD, such as antispasmodics, laxatives, anti-inflammatory agents, and immunosuppressants, are often associated with adverse side effects, leading to increased interest in alternative therapeutic approaches, including probiotic interventions [4,5]. When administered in the right dosages, probiotics-live microorganisms-can enhance a host's immune system and gut microbiome [6].

The aim of this systematic review is to critically evaluate the effectiveness of probiotic interventions in managing symptoms of IBS and IBD in adult patients. By synthesizing evidence from randomized controlled trials (RCTs) and observational studies, this review seeks to clarify the role of probiotics in symptom alleviation and overall quality of life for individuals suffering from these gastrointestinal disorders.

## Review

Methods

In order to evaluate the effectiveness of probiotic therapies in adult populations affected by IBS or IBD, a thorough systematic review was carried out between May 15, 2024, and May 30, 2024. The search encompassed the databases PubMed, Cochrane Library, and Google Scholar, as well as the clinical trial registries ClinicalTrials.gov and International Standard Randomized Controlled Trial Number (ISRCTN). The search strategy was developed using a combination of relevant keywords, Boolean operators (AND, OR, and NOT), and MeSH terms related to IBS, IBD, probiotics, and efficacy (Table 1).

**Table 1:** Search Strategy ISRCTN: International Standard Randomized Controlled Trial Number

Database	Search strategy
PubMed/Medline	( "Irritable Bowel Syndrome"[Mesh] OR ("Irritable Bowel Syndrome" OR ''IBS'' OR ''IBS'' [Text Word] OR "Irritable Colon" OR "Colon, Irritable"[Text Word]) ) OR ("Inflammatory Bowel Diseases"[Mesh] OR "Inflammatory Bowel Disease" OR IBD'' OR ''IBD'' [Text Word] OR "Ulcerative Colitis" OR "Crohn's Disease"[Text Word]) OR "Gastrointestinal Tract"[Mesh] AND ( "Probiotics"[Mesh] OR ("Probiotics*" OR "Gut Microbiome" OR "Lactobacillus" OR "Bifidobacterium" OR "Saccharomyces boulardii"[Text Word]) ) AND ( "treatment outcome"[Text Word] OR "efficacy"[Text Word] OR "effectiveness"[Text Word] OR "symptoms"[Text Word] OR "quality of life" [Text Word])
Cochrane Library	MeSH descriptor: [Irritable Bowel Syndrome] explode all trees; ("irritable bowel syndrome"):ti,ab,kw; ("irritable colon syndrome"):ti,ab,kw OR MeSH descriptor: [Inflammatory Bowel Diseases] explode all trees; ("inflammatory bowel disease"):ti,ab,kw AND MeSH descriptor: [Probiotics] explode all trees; ("probiotic"):ti,ab,kw AND MeSH descriptor: [Quality of Life] this term only; ("quality" NEAR/3 "life"):ti,ab,kw; ("QOLI"):ti,ab,kw
Google Scholar	allintitle: Probiotics AND Irritable bowel syndrome OR Inflammatory bowel disease -review
ClinicalTrials.gov	Completed Studies | Studies With Results | Interventional Studies | Irritable Bowel Syndrome | Probiotics | Adult Completed Studies | Observational Studies | Irritable Bowel Syndrome | Probiotics | Adult Inflammatory Bowel Disease | probiotics | Completed studies | Adult (18 - 64) | Interventional studies | Studies with results Inflammatory Bowel Disease | probiotics | Completed studies | Adult (18 - 64) | Observational studies | Studies with results
ISRCTN registry	Irritable bowel syndrome OR Inflammatory bowel disease AND Probiotics AND Efficacy

This systematic review adhered to the Preferred Reporting Items for Systematic Reviews and Meta-Analyses (PRISMA) 2020 guidelines [7] to ensure the comprehensive identification of relevant studies, while following the inclusion and exclusion criteria.

Inclusion Criteria

The inclusion criteria include adult patients aged 18 years and older diagnosed with IBS or IBD; studies where patients are given probiotics alone or studies comparing probiotics with placebo; studies published in English; study designs such as RCTs, non-randomized clinical trials, case-control, cross-sectional studies, or cohort studies; and studies reporting at least one relevant outcome measure such as symptom improvement, pain score, bloating score, stool consistency, and quality of life measures.

Exclusion Criteria

The exclusion criteria include participants diagnosed with gastrointestinal conditions other than IBS or IBD; studies involving interventions that include probiotics combined with other treatments for IBS or IBD; studies published in languages other than English; review articles, case reports, and case series, or those involving animals or pregnant or breastfeeding subjects; and studies lacking any clinical outcome (risk, benefit, or no significance) with the use of probiotics.

Data Extraction and Quality Assessment

The keywords employed during the search included "IBS", "IBD", "Probiotics", and "Efficacy". This comprehensive approach aimed to capture the published literature evaluating the efficacy of probiotic interventions in adult patients with IBS or IBD. Duplicate studies were removed using EndNote (Clarivate, London, UK), and the final search terms and combinations were documented for reference in PubMed and other databases.

The systematic search and review process was carried out rigorously to gather the evidence currently available on the use of probiotics in the targeted patient populations. This methodical approach ensured a comprehensive evaluation of the existing research in order to arrive at relevant conclusions about the efficacy of probiotic treatments for IBS and IBD in adult patients.

To ensure uniformity and thoroughness, a pre-defined checklist was used during the data extraction process by two authors, AM and GS. Any conflicts or discrepancies that arose during this process were resolved through discussion and consensus with a third author, IM. Using suitable tools, such as the Cochrane Risk of Bias (RoB 2) tool [8] for randomized trials and the Newcastle-Ottawa Scale (NOS) [9] for cohort studies, the overall methodological quality and potential risk of bias were assessed in the selected research. This thorough quality assessment made it possible to evaluate the contained evidence in a robust manner.

By adhering to established systematic review guidelines and utilizing validated quality appraisal instruments, the review provided a rigorous and comprehensive synthesis of the existing literature on the efficacy of probiotic interventions in adult populations with IBS or IBD. The rigorous procedures for collecting data and quality assessment guaranteed the authenticity and dependability of the review's conclusions, which can direct future research in this crucial field and influence healthcare choices.

Results

Database and Register Search Results

**Figure 1:**
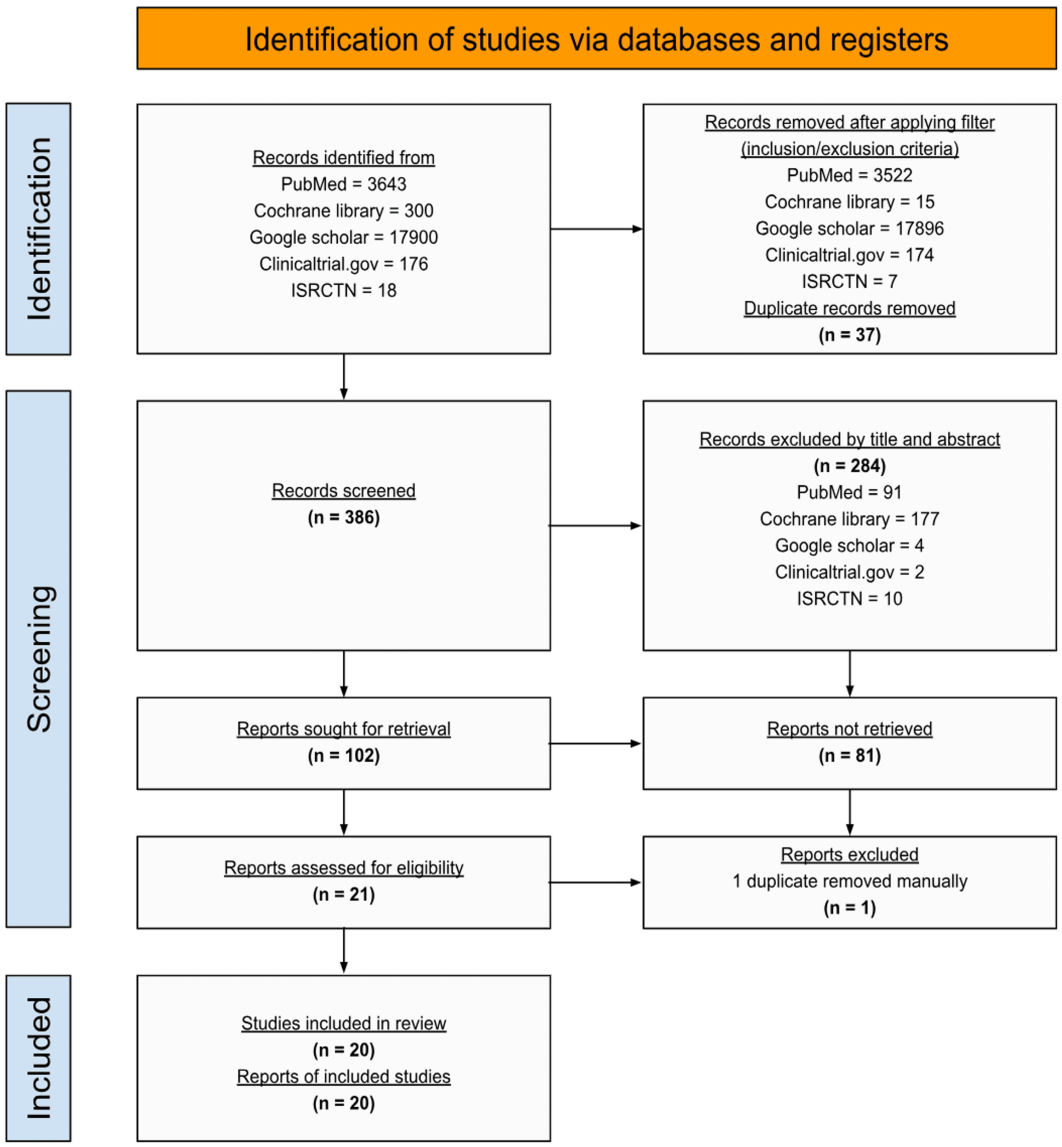
The PRISMA Flow Diagram Details the Screening and Selection Process PRISMA: Preferred Reporting Items for Systematic Reviews and Meta-Analyses; ISRCTN: International Standard Randomized Controlled Trial Number

Using the search approach described in the methodology section, a total of 21,843 studies were found across the databases PubMed, Cochrane Library, and Google Scholar, along with an additional 194 studies from Clinicaltrials.gov and ISRCTN. After applying inclusion and exclusion criteria filters and removing duplicates using EndNote, 386 unique studies remained. These studies were then screened by title and abstract, resulting in 102 studies. Subsequently, we checked for the retrieval of the full texts and identified 21 studies. During the eligibility assessment, one duplicate was found and removed manually. Therefore, this review contained a total of 20 reviews, here summarized in the PRISMA flow diagram (Figure 1).

Risk of Bias Assessment

The risk of bias in the included studies (n = 20) was evaluated utilizing tools appropriate for each study design: the RoB 2 for RCTs [8] and the NOS for cohort studies [9] (see Table 2). Different levels of bias were found in each study, based on the assessment. The majority of the RCTs were found to have a low risk of bias, but a small number were deemed to have a moderate risk because of issues with the randomization and allocation concealment methods, as well as possible bias resulting from incomplete outcome data. The NOS tool evaluated the two included cohort studies and found them to be of moderate quality.

**Table 2:** Summary of Study Design, Quality Assessment, and Inclusion Decisions for Analyzed Studies RCT: randomized controlled trial

Study	Design	Title	Quality assessment tool	Assessment summary	Inclusion decision
Begtrup et al. (2013) [10]	RCT	Long-term treatment with probiotics in primary care patients with irritable bowel syndrome--a randomised, double-blind, placebo controlled trial	Cochrane Risk of Bias 2 tool	Some concerns of bias (high dropout rate (27%) and potential bias due to missing outcome data)	Included
Dapoigny et al. (2012) [11]	RCT	Efficacy and safety profile of LCR35 complete freeze-dried culture in irritable bowel syndrome: a randomized, double-blind study	Cochrane Risk of Bias 2 tool	Some concerns of bias (unclear randomization and allocation concealment procedures, high dropout rate, and potential bias due to missing outcome data)	Included
Guglielmetti et al. (2011) [12]	RCT	Randomised clinical trial: Bifidobacterium bifidum MIMBb75 significantly alleviates irritable bowel syndrome and improves quality of life--a double-blind, placebo-controlled study	Cochrane Risk of Bias 2 tool	Low risk of bias	Included
Guyonnet et al. (2007) [13]	RCT	Effect of a fermented milk containing Bifidobacterium animalis DN-173 010 on the health-related quality of life and symptoms in irritable bowel syndrome in adults in primary care: a multicentre, randomized, double-blind, controlled trial	Cochrane Risk of Bias 2 tool	Low risk of bias	Included
Kajander et al. (2005) [14]	RCT	A probiotic mixture alleviates symptoms in irritable bowel syndrome patients: a controlled 6-month intervention	Cochrane Risk of Bias 2 tool	Low risk of bias	Included
Kruis et al. (2012) [15]	RCT	A double-blind placebo-controlled trial to study therapeutic effects of probiotic Escherichia coli Nissle 1917 in subgroups of patients with irritable bowel syndrome	Cochrane Risk of Bias 2 tool	Low risk of bias	Included
Roberts et al. (2013) [16]	RCT	A randomised controlled trial of a probiotic 'functional food' in the management of irritable bowel syndrome	Cochrane Risk of Bias 2 tool	Some concerns of bias (unclear reporting of randomization and allocation concealment, high dropout rate, and potential bias due to missing outcome data)	Included
Simrén et al. (2010) [17]	RCT	Clinical trial: the effects of a fermented milk containing three probiotic bacteria in patients with irritable bowel syndrome-a randomized, double-blind, controlled study	Cochrane Risk of Bias 2 tool	Low risk of bias	Included
Kato et al. (2004) [18]	RCT	Randomized placebo-controlled trial assessing the effect of bifidobacteria-fermented milk on active ulcerative colitis	Cochrane Risk of Bias 2 tool	Low risk of bias	Included
Catinean et al. (2019) [19]	RCT	Bacillus spp. spores-a promising treatment option for patients with irritable bowel syndrome	Cochrane Risk of Bias 2 tool	Some concerns of bias (concerns about the randomization and allocation concealment process, and potential bias due to missing outcome data)	Included
Lewis et al. (2020) [20]	RCT	Efficacy of Lactobacillus paracasei HA-196 and Bifidobacterium longum R0175 in alleviating symptoms of irritable bowel syndrome (IBS): a randomized, placebo-controlled study	Cochrane Risk of Bias 2 tool	Low risk of bias	Included
Lorenzo-Zúñiga et al. (2014) [21]	RCT	I.31, a new combination of probiotics, improves irritable bowel syndrome-related quality of life	Cochrane Risk of Bias 2 tool	Low risk of bias	Included
Martoni et al. (2020) [22]	RCT	Lactobacillus acidophilus DDS-1 and Bifidobacterium lactis UABla-12 improve abdominal pain severity and symptomology in irritable bowel syndrome: randomized controlled trial	Cochrane Risk of Bias 2 tool	Low risk of bias	Included
Pinto-Sanchez et al. (2017) [23]	RCT	Probiotic Bifidobacterium longum NCC3001 reduces depression scores and alters brain activity: a pilot study in patients with irritable bowel syndrome	Cochrane Risk of Bias 2 tool	Low risk of bias	Included
Sabaté and Iglicki (2022) [24]	Cohort	Effect of Bifidobacterium longum 35624 on disease severity and quality of life in patients with irritable bowel syndrome	Newcastle-Ottawa Scale	Moderate quality: selection (3/4), comparability (1/2), outcome (2/3)	Included
Bjarnason et al. (2019) [25]	RCT	A randomised, double-blind, placebo-controlled trial of a multistrain probiotic in patients with asymptomatic ulcerative colitis and Crohn's disease	Cochrane Risk of Bias 2 tool	Low risk of bias	Included
Matsuoka et al. (2018) [26]	RCT	Efficacy of Bifidobacterium breve fermented milk in maintaining remission of ulcerative colitis	Cochrane Risk of Bias 2 tool	Low risk of bias	Included
Oh et al. (2021) [27]	Cohort	Therapeutic potential of Escherichia coli Nissle 1917 in clinically remission-attained ulcerative colitis patients: a hospital-based cohort study	Newcastle-Ottawa Scale	Moderate quality: selection (3/4), comparability (1/2), outcome (2/3)	Included
Yılmaz et al. (2019) [28]	RCT	Effect of administering kefir on the changes in fecal microbiota and symptoms of inflammatory bowel disease: a randomized controlled trial	Cochrane Risk of Bias 2 tool	Low risk of bias	Included
Yoshimatsu et al. (2015) [29]	RCT	Effectiveness of probiotic therapy for the prevention of relapse in patients with inactive ulcerative colitis	Cochrane Risk of Bias 2 tool	Low risk of bias	Included

Since the review did not reveal any high-risk or low-quality research that would have justified exclusion, all studies were incorporated in the final analysis to ensure higher confidence in its overall findings. By carefully evaluating the methodological quality of the included studies, the review ensured that the final synthesis was based on evidence with an appropriate level of internal validity, strengthening the reliability and confidence in the findings.

Summary of Studies Evaluating Probiotics for IBS

A growing number of studies are looking into the potential benefits of probiotics for IBS. Table 3 summarizes the findings from 13 RCT studies and one open-label observational study looking into probiotic use for IBS. All RCTs were double-blinded except for Catinean et al. [19] and Sabaté and Iglicki [24].

**Table 3:** Summary of Studies Evaluating Probiotics for Irritable Bowel Syndrome RCT: randomized controlled trial; IBS: irritable bowel syndrome; IBS-C: irritable bowel syndrome with constipation; IBS-D: irritable bowel syndrome with diarrhea; HRQoL: health-related quality of life; CI: confidence interval; s.d.: standard deviation; FODMAP: fermentable, oligo-, di-, monosaccharide and polyol; VSI: Visceral Sensitivity Index; APS-NRS: Abdominal Pain Severity-Numeric Rating Scale; IBS-SSS: IBS Symptom Severity Scale; HAD: Hospital Anxiety and Depression

Authors/year of publication	Type of study	Number of participants	Age	Intervention/exposure	Control group	Duration of treatment/follow-up	Results
Begtrup et al. (2013) [10]	RCT double-blinded	131	18-50 years	Probiotic mixture strains: Lactobacillus paracasei ssp. paracasei F19, Lactobacillus acidophilus La5, and Bifidobacterium Bb12. Dosage: two capsules twice a day; colony-forming units (CFUs) per capsule: 1.3 × 10^10^	Placebo	Six months of treatment and six months of follow-up	Responder rate: probiotic (52%, 35/67) vs. placebo (41%, 26/64) (p = 0.18, not significant). Symptoms: no significant difference between groups in symptom changes after treatment
Dapoigny et al. (2012) [11]	RCT double-blind pilot study	50	18-70 years	LCR35 at a minimum daily dose of 6 × 10^8^ CFUs	Placebo	Four weeks of treatment and two weeks of follow-up	Abdominal pain: test drug (-13.1 ± 20.5) and placebo (-11.9 ± 27.5) showed similar improvements. Diarrhea-predominant IBS: test drug (-18.4 ± 26.3, 36% decrease) vs. placebo (-0.1 ± 26.5). Overall IBS severity: no significant difference (p-value = 0.9692 at the end of treatment, 0.8829 at follow-up) with similar decreases observed throughout the study (test drug: -63.2 ± 100.6 at the end, -40.6 ± 110.1 at follow-up; placebo: -64.3 ± 95.9 at the end, -36.0 ± 109.5 at follow-up)
Guglielmetti et al. (2011) [12]	RCT double-blinded	122	18-68 years	MIMBb75 once a day for four weeks	Placebo	Four weeks of treatment, follow-up after two, six, and eight weeks (end of wash-out phase)	Global IBS symptoms: MIMBb75 significantly reduced symptoms (-0.88 points, 95% CI: -1.07; -0.69) compared to placebo (-0.16 points, 95% CI: -0.32; 0.00) (p < 0.0001). Specific symptoms: MIMBb75 significantly improved pain/discomfort, distension/bloating, urgency, and digestive disorders. Quality of life: SF12 scores showed significant improvement in the MIMBb75 group. Responder rates: adequate relief: 47% (MIMBb75) vs. 11% (placebo) (p < 0.0001). Overall responders: 57% (MIMBb75) vs. 21% (placebo) (p = 0.0001). Safety: MIMBb75 was well tolerated with no difference in adverse events compared to placebo
Guyonnet et al. (2007) [13]	RCT double-blinded	274	18-65 years	Product: fermented milk; probiotic strain: Bifidobacterium animalis DN-173 010. Probiotic dosage: 1.25 x 10^10^ CFUs per serving. Yogurt starter cultures: Streptococcus thermophilus (S. thermophilus), Lactobacillus bulgaricus (L. bulgaricus). Dosage: 1.2 x 10^9^ CFU/serving (combined)	Heat-treated yogurt containing non-living bacteria (<10^4^ CFU/pot)	Six weeks of treatment period. HRQoL and digestive symptoms were assessed after three and six weeks on an intention-to-treat population of 267 subjects	HRQoL discomfort score: improved in both groups (probiotic and control) over time, but a significantly larger proportion of the probiotic group (65.2%) achieved a meaningful improvement at week three (p < 0.005). Probiotic group: showed additional benefits in reducing bloating (p = 0.03) and increasing stool frequency for those with constipation
Kajander et al. (2005) [14]	RCT double-blinded	103	20-65 years	Type: probiotic mixture. Strains: (equal amount of each strain included) Lactobacillus rhamnosus GG, Lactobacillus rhamnosus LC705, Bifidobacterium breve Bb99, Propionibacterium freudenreichii ssp. shermanii JS. Dosage: one capsule daily. Total CFU per day: 8-9 × 10^9^ CFUs	Placebo capsules	Six months of treatment period and follow-up after every one month	Total IBS symptom score (abdominal pain + distension + flatulence + borborygmi): probiotic group showed a significantly lower score (7.7 points lower, 95% CI: -13.9 to -1.6) compared to placebo (p = 0.015). This translates to a median reduction of 42% in symptoms for the probiotic group compared to 6% for placebo. Specific symptoms: only borborygmi (gurgling sounds in the stomach) showed a significant improvement (p = 0.008) in the probiotic group. Other symptoms showed a non-significant positive trend
Kruis et al. (2012) [15]	RCT double-blinded	120	18-65 years	Strain: Escherichia coli strain Nissle 1917. Dosage range: 2.5 billion-25 billion CFUs per day	Placebo	12 weeks of treatment period and follow-up every four weeks	Responder rate: probiotic EcN showed a higher responder rate than placebo, but only significantly after 10 and 11 weeks (20.0% and 18.3% difference, respectively). Best responders: patients with prior gastroenteritis or antibiotic use showed the greatest response (45.7% difference). No significant differences were observed in other subgroups. Both groups had similar side effects and tolerability
Roberts et al. (2013) [16]	RCT double-blinded	179	18-65 years	Probiotic strain: Bifidobacterium lactis CNCM I-2494. Probiotic dosage: 1.25 x 10^10^ CFUs per serving (cup). Yogurt starter cultures: Streptococcus thermophilus (S. thermophilus) and Lactobacillus bulgaricus (L. bulgaricus) (combined). Dosage: 1.2 x 10^9^ CFU/serving (cup)	Milk-based non-fermented dairy product without probiotics and with similar lactose content to the test product	12-week treatment period and follow-up visits after four, eight, and 12 weeks	Adequate relief: no significant difference at week four (57% probiotic vs. 53% placebo, p = 0.71). The probiotic group showed a lower responder rate compared to placebo at week eight (46% vs. 68%, p = 0.03) and continued till week 12. Limitations: high dropout rates might have impacted the results
Simrén et al. (2010) [17]	RCT double-blinded	74	26- 58 years	400-mL fermented milk containing Lactobacillus paracasei ssp. paracasei F19, Lactobacillus acidophilus La5, and Bifidobacterium lactis Bb12 (Cultura)	Placebo	Eight weeks of treatment period and follow-up every two weeks	Responder rate: no significant difference between the probiotic group (38%) and the control group (27%) (p = 0.3). IBS severity: both groups showed significant improvement in symptoms, with the probiotic group experiencing a greater initial improvement in the first two weeks (not statistically significant though). Overall: no clear benefit of the probiotic compared to control was observed. However, there was a hint of a possible early positive effect with the probiotic
Catinean et al. (2019) [19]	RCT open-label study	90	18-75 years	Group 1 (G1): rifaximin (1,200 mg daily) for 10 days followed by a nutraceutical agent (probiotic + prebiotics + vitamins) for 54 days. Group 2 (G2): spore-based probiotic formulation with five Bacillus spp. (MegaSporeBiotic) for 34 days. Group 3 (G3): rifaximin (1,200 mg daily) for 10 days followed by a low-FODMAP diet for 54 days	No control group	Four weeks of treatment period and two weeks of follow-up	IBS severity: all groups improved over time, but the MegaSporeBiotic group (G2) had a significantly lower IBS severity score compared to the rifaximin + nutraceutical group (G1) at week four (p = 0.038). There was no significant difference between G2 and the rifaximin + low-FODMAP diet group (G3). Quality of life: the MegaSporeBiotic group (G2) showed significantly better scores in aspects like general health and physical functioning compared to both G1 and G3 at weeks four and eight (p < 0.05). Rectal volume sensation: all groups improved over time, but the MegaSporeBiotic group (G2) demonstrated a greater improvement in "first sensation" compared to the other groups at week four (p = 0.016 & 0.001)
Lewis et al. (2020) [20]	RCT double-blinded	285	18 years and older	Strains: the capsules contain either Bifidobacterium longum (B. longum) (lot numbers: NH131210-1VB and NH151104-ICP) or Lactobacillus paracasei (L. paracasei) (lot numbers: NH131217-1VB and NH151106-ICP). Dosage: each capsule contains 10 billion CFUs of probiotic bacteria	Placebo	Eight weeks of treatment period and two weeks of follow-up	IBS severity (all groups): reduced scores (20%-30% at week eight), but no significant difference between probiotics and placebo. IBS-C: L. paracasei increased spontaneous bowel movements (weeks four & eight). IBS-D: L. paracasei decreased spontaneous bowel movements (week eight, details on significance not mentioned) and improved stool consistency (weeks four & eight). Psychological: both probiotics improved emotional well-being and social functioning. Microbiome: no significant changes observed in specific bacteria
Lorenzo-Zúñiga et al. (2014) [21]	Multicenter RCT double-blinded	84	20-70 years	Probiotic combination: two Lactobacillus plantarum strains (CECT7484 & CECT7485), one Pediococcus acidilactici strain (CECT7483). Dosages: high dose: 1-3 x 10^10^ CFU/capsule throughout the study. Low dose: 3-6 x 10^9^ CFU/capsule throughout the study	Placebo	Six weeks of treatment	IBS-related quality of life (IBS-QoL): probiotic significantly improved IBS-QoL compared to placebo (p = 0.008). Probiotic groups saw a greater increase in IBS-QoL scores: high dose: +18 ± 3 points (p = 0.041 vs. placebo); low dose: +22 ± 4 points (p = 0.023 vs. placebo); placebo: +9 ± 3 points. Gut-specific anxiety (VSI score): probiotic treatment significantly reduced gut-specific anxiety: high dose: -10 ± 2 points (p < 0.05 vs. placebo); low dose: -14 ± 2 points (p < 0.05 vs. placebo); placebo: -7 ± 1 point. Symptom relief & safety: no significant differences in symptom relief between groups. No adverse reactions reported with probiotic consumption
Martoni et al. (2020) [22]	Multicenter, RCT double-blinded	330	18-70 years	Probiotic intervention: Lactobacillus acidophilus DDS-1 (1 x 10^10^ CFU/day), Bifidobacterium animalis subsp. lactis UABla-12 (1 x 10^10^ CFU/day)	Placebo	Six weeks of treatment and follow-up after six weeks	Abdominal pain: both probiotics significantly decreased pain scores (APS-NRS) vs. placebo (p < 0.001). Overall symptoms: both strains reduced overall IBS symptom severity (IBS-SSS) scores (p < 0.001), including subscores for pain, bloating, bowel habits, and quality of life. Stool consistency: probiotics normalized stool consistency compared to placebo
Pinto-Sanchez et al. (2017) [23]	RCT double-blinded pilot study	44	26-58 years	Probiotic: 42 sachets of spray-dried Bifidobacterium longum (BL)	Placebo	Six weeks of treatment and follow-up after 0.6 and 10 weeks	(Week six) depression: BL group: 14/22 (64%) patients had a ≥2-point reduction in depression scores (HAD scale) vs. 7/22 (32%) in the placebo group (p = 0.04). BL had no significant effect on anxiety symptoms. Quality of life: the BL group showed a mean increase in quality of life score compared to placebo. Brain imaging (fMRI): BL treatment reduced responses to negative emotional stimuli in brain areas associated with emotion processing (amygdala, fronto-limbic regions) compared to placebo. Week 10 follow-up: depression scores remained lower in the BL group
Sabaté and Iglicki (2022) [24]	Prospective, open-label, multicenter, observational study	233	37-66 years	B. longum 35624 (10^9^ CFUs) per day for 30 days	No control group	30 days of treatment and follow-up after 30 days	IBS severity score: 208 vs. 303 (baseline) (p < 0.001). 57% of patients improved or achieved remission. Quality of life score: 68.8 vs. 60.2 (baseline) (p < 0.001); 63.8% of patients were satisfied

The study designs varied, with most being RCTs with a double-blind approach, where neither the researchers nor the participants were aware of who received the probiotic or the placebo. However, one study was an open-label observational study, where both the participants and the researchers were aware that everyone received the probiotic, with no placebo group.

The studies' sample sizes varied from 44 to 330 people, with adults aged 18 to 70 making up the majority of the participant group. The interventions used different probiotic strains and formulations, with varying dosages across the studies.

Regarding the control groups, all RCTs had a placebo group, except Catinean et al. [19], and one open-label observational study, Sabaté and Iglicki [24], did not have a control group. There were follow-up intervals along with the duration of the four- to six-month course of treatment.

The primary outcomes often included scores for the intensity of IBS symptoms and overall life satisfaction in relation to IBS. The secondary outcomes varied but could include specific symptoms (e.g., bloating, pain, and diarrhea), anxiety, depression, and stool consistency.

Summary of Studies Evaluating Probiotics for IBD

Researchers have been exploring the probiotics' possible therapeutic use in the treatment of IBD [18,25-29]. Table 4 compiles the results of five RCTs and one observational, retrospective cohort study that assessed the use of probiotics for IBD.

**Table 4:** Summary of Studies Evaluating Probiotics for Inflammatory Bowel Disease RCT: randomized controlled trial; FCAL: fecal calprotectin; UC: ulcerative colitis; CD: Crohn's disease; ESR: erythrocyte sedimentation rate; CRP: C-reactive protein; CFU: colony-forming units

Authors/year of publication	Type of study	Number of participants	Age	Intervention/exposure	Control group	Duration of treatment/follow-up	Results
Kato et al. (2004) [18]	RCT double-blinded pilot study	20	Bifidobacteria-fermented milk (BFM) group mean age = 30.2 years. Placebo mean age = 33.7 years	100 mL/day of BFM	Placebo	12 weeks of treatment and follow-up after every four weeks	Both groups improved, but the fermented milk group showed significantly greater improvement in clinical symptoms, gut lining appearance (endoscopy), and tissue samples (histology). No side effects were reported in either group
Bjarnason et al. (2019) [25]	RCT double-blinded	142	18-70 years	1 mL/kg/day probiotic	Placebo	Four weeks of treatment	FCAL, a marker of gut inflammation, showed a significant decrease (p < 0.015) in UC patients taking probiotics compared to placebo. Quality of life: no significant changes seen. No side effects
Matsuoka et al. (2018) [26]	RCT double-blinded	195	20-70 years	One pack of BFM fermented milk per day (Bifidobacterium breve strain Yakult (10 billion bacteria) and Lactobacillus acidophilus (1 billion bacteria))	Placebo	48 weeks of treatment and follow-up at four, 12, 24, and 36	Relapse rates: no significant difference between BFM and placebo (p = 0.643). Gut bacteria: Bifidobacterium levels decreased in both groups before relapse, regardless of treatment. Side effects: minor and infrequent (abdominal bloating, stress, and body odor)
Oh et al. (2021) [27]	Observational, retrospective study, cohort study	94	23-76 years	Escherichia coli Nissle 1917 capsule, one capsule twice daily (2.5 × 10^9^ viable bacteria per capsule) + standard treatment	No control group	Three months of treatment	FCAL: no significant change (p = 0.653). Partial Mayo Score: significant improvement (p = 0.025). Body weight & BMI: significant increase (p = 0.001 & <0.001). Adverse events: one patient (1.1%) experienced a serious UC flare-up. Fourteen patients (14.9%) discontinued due to side effects within three months
Yılmaz et al. (2019) [28]	RCT open-label	45	Treatment group: 24 to 65 years. Control group: 21 to 66 years	400 mL/day kefir twice a day, which contains a total of 2.0 × 10^10^ CFU/mL viable Lactobacillus bacteria	No placebo product was provided. The control group did not consume any kefir or other probiotic-containing products	Four weeks of treatment	Gut bacteria: fecal levels of Lactobacillus bacteria significantly increased in the treatment group (p = 0.001 for UC, p = 0.005 for CD). Significant decrease in inflammatory markers (ESR & CRP). Reduced bloating scores in the last two weeks (p = 0.012). Increased feelings of well-being (p = 0.032)
Yoshimatsu et al. (2015) [29]	RCT double-blinded	60	Bio-three group: mean age: 44.8 ± 13.8 years. Placebo group: mean age: 42.9 ± 15.9 years	2 mg of lactomin (Streptococcus faecalis T-110), 10 mg of Clostridium (Clostridium butyricum TO-A), and 10 mg of Bacillus (Bacillus mesentericus TO-A), 3 tabs x 3 times/day	Placebo	12 months of treatment, follow-up every month	Relapse rates: significantly lower relapse rates in the Bio-Three group compared to placebo at three months (0% vs. 17.4%, p = 0.036). Trend toward lower relapse rates in the Bio-Three group at later time points, but not statistically significant. Remission rate at 12 months: 69.5% (Bio-Three) vs. 56.6% (placebo), not statistically significant (p = 0.248). Fecal flora clusters: the study suggests a potential link between gut bacteria composition and response to probiotics, but more research is needed

The study designs varied, with the majority being double-blind RCTs [18,25,26,29]. However, one study was an open-label observational cohort design, where all participants received the probiotic intervention [27]. There were 556 participants with an age range spanning from 18 to 76 years throughout the investigations.

The probiotic interventions included bifidobacteria-fermented milk [18,26], a multistrain probiotic [25], Escherichia coli Nissle 1917 capsules [27], kefir containing Lactobacillus bacteria [28], and a combination probiotic known as Bio-Three [29]. In RCTs, the control groups consisted of placebo; in contrast, no control group was available in the observational study. The course of treatment ranged from four weeks to 12 months, with various follow-up periods.

The studies evaluated a range of outcomes to assess the potential benefits of probiotic interventions for IBD. These included clinical and endoscopic activity indices [18,27], histological scores [18,26], fecal calprotectin levels as a marker of gut inflammation [25,27], quality of life measures [25,28], gut microbiome composition [26,28], and the safety and tolerability of the probiotic supplements [18,26,27]. The varied outcome measures provided perceptions of the potential effects of probiotics on disease activity, intestinal inflammation, and patient-reported outcomes, which are crucial for understanding the clinical relevance of probiotic therapies for IBD management.

Discussion

This systematic study aims to evaluate the efficacy of probiotic therapy for adult patients with IBS and IBD.

Efficacy of Probiotics in IBS

The possible effectiveness of probiotic therapies for the therapy of IBS in adult patients is revealed by the systematic analysis of 13 RCTs and one observational research. The RCTs, most of which employed a double-blind design, assessed a range of probiotic strains and formulations, such as fermented dairy products that include probiotics and single- and multistrain probiotics. The trials assessed a wide range of outcome factors, such as the intensity of symptoms associated with IBS, the overall standard of living related to IBS, specific IBS symptoms (including diarrhea, bloating, and stomach pain), and psychological traits (like anxiety and depression).

The results from the RCTs present a mixed picture of the effectiveness of probiotics in treating IBS. While some studies reported notable improvements in IBS symptom scores, standard of living [19,23], and specific symptoms like abdominal pain and bloating in the probiotic groups compared to placebo [12,14,21,22], other trials did not find any statistically significant changes between the probiotic and placebo groups [11,15-17,20]. The open-label observational study, which lacked a control group, also reported improvements in IBS severity and quality of life with the probiotic intervention [24].

Nonetheless, some studies did demonstrate promising results, particularly in terms of improving specific IBS symptoms, such as abdominal pain, bloating, and stool consistency, as well as enhancing quality of life and psychological well-being [12,21,22]. These findings suggest that certain probiotic interventions may be beneficial for subgroups of IBS patients, underscoring the importance of further research to identify the strains, dosages, and patient characteristics that are most probable to gain from probiotic treatment.

Similar mixed findings have been reported in other systematic reviews and meta-analyses on this topic. For instance, Xie et al.'s network meta-analysis from 2023 found that a number of probiotic strains and combinations significantly outperformed placebo in terms of improving several IBS outcomes [30]. According to Zhang et al.'s systematic review and network meta-analysis, Bacillus coagulans showed the highest likelihood of being the finest types of probiotics for enhancing the alleviation of IBS symptoms [31].

Consistent with our findings, a more recent systematic review by Wilkins et al. also reported mixed results [32]. Seven (63.6%) of the 11 research that made up their evaluation found that probiotic supplementation significantly reduced IBS symptoms when compared to a placebo, whereas the remaining four (36.4%) studies found no discernible changes. The authors report that the beneficial effects were more obvious in trials using multistrain probiotic supplements for a minimum of eight weeks [32].

The observed variability in the study findings may be attributed to several factors, including the heterogeneity of the probiotic strains and formulations used, the varying study durations, and the diverse patient populations enrolled. Additionally, the inherent complexity and multifactorial nature of IBS, with its various subtypes (e.g., IBS-D and IBS-C), may contribute to the mixed results across the different systematic reviews and meta-analyses.

Efficacy of Probiotics in IBD

The systematic review of five RCTs and one observational cohort study evaluated the efficacy of probiotic interventions in patients with IBD, including CD and UC. The RCTs employed a double-blind design, with the exception of one RCT, which was an open-label study, that assessed various probiotic formulations, such as bifidobacteria-fermented milk, a multistrain probiotic, and a combination probiotic known as Bio-Three [28]. The studies evaluated a range of outcomes, including clinical and endoscopic disease activity, histological scores, fecal calprotectin as a marker of gut inflammation, quality of life, and changes in the gut microbiome.

The findings from the RCTs were mixed. While one study noted a notable decrease in clinical symptoms, endoscopic appearance, and histological scores in the bifidobacteria-fermented milk group compared to placebo [18], other studies did not discover any appreciable variations in the relapse rates or other outcomes between the probiotic and placebo groups [25,26,29].

An open-label RCT by Yılmaz et al. investigated the effects of kefir, a fermented milk drink containing Lactobacillus, in adults with UC [28]. This study, while lacking a control group, suggests a potential benefit. Kefir consumption for four weeks led to improvements in gut bacteria, reduced inflammation, and enhanced well-being.

An observational cohort study, also lacking a control group, investigated the use of Escherichia coli Nissle 1917 probiotics in UC patients. This study revealed improvements in the disease activity score (Partial Mayo Score) and weight gain, but no significant changes in fecal calprotectin, a marker of inflammation [27].

The heterogeneity of the probiotic strains, formulations, and study designs, as well as the underlying complexity of IBD pathogenesis, may contribute to the variable results observed across the studies. It is important to remember that probiotic therapies were typically well tolerated, with very mild side effects recorded, even in studies where no significant changes were seen between the probiotic and control groups. The observed trends toward improved clinical outcomes, such as reduced relapse rates and enhanced remission maintenance in some studies, suggest that specific probiotic strains or combinations may have the potential to benefit subsets of IBD patients [18,29].

In contrast to the mixed findings we observed in our IBS systematic review, the available evidence on the use of probiotics for IBD, including CD and UC, appears to be more promising, particularly for UC. A recent umbrella review and updated meta-analysis by Estevinho et al. discovered that the use of probiotics was associated with significantly higher odds of achieving clinical cessation in UC patients (OR 2.00, 95% CI 1.28-3.11), though this effect was not seen in CD [33]. Additionally, probiotics were found to decrease the chances of clinical relapse in UC patients and those with relapsing pouchitis. Notably, when it comes to UC remission and recurrence prevention, the De Simone probiotic formulation continuously performs better than alternative probiotic therapies.

On the contrary, a Cochrane review by Limketkai et al. on probiotics for the induction of remission in CD found that, when compared to a placebo, there is currently very little data to support the safety or effectiveness of probiotics, highlighting the need for more carefully planned studies in this field [34]. Similarly, a previous systematic review and meta-analysis by Derwa et al. stated that probiotics' effectiveness in treating CD is still unknown, while they might work just as well as aminosalicylic acids (5-ASAs) to stop relapses in quiescent UC [35]. These findings indicate that the potential benefits of probiotics may be more evident in UC compared to CD, which contrasts with the more mixed results we observed across the IBS systematic reviews.

Comparison of Probiotic Efficacy in IBS and IBD

When comparing the efficacy of probiotics in the management of IBS and IBD, several observations can be made. Numerous outcome measures were applied in the research on IBS and IBD; however, considerable overlap was observed. Clinical symptoms, standard of living, and safety/tolerability were evaluated for both illnesses. IBD research also assessed fecal calprotectin levels and other indicators of illness activity, such as endoscopic and histological scores.

Probiotic therapies were found to have a variety of beneficial effects for IBS and IBD, with some trials finding statistically significant differences from the placebo or control groups, while other trials found no noticeable improvements. The heterogeneity of the probiotic strains, formulations, and study methodologies may be the cause of this heterogeneity.

Despite the mixed overall findings, several studies in both IBS and IBD suggested that particular strains of probiotics or combinations may be beneficial for particular subgroups of patients. For example, some IBS studies found improvements in abdominal pain, bloating, and consistency of stool, while some IBD studies reported reduced relapse rates and enhanced remission maintenance.

Across the studies on both IBS and IBD, the probiotic interventions were usually well tolerated, with only mild adverse events reported in a few instances. This indicates that probiotic therapies may be a safe and feasible option for consideration in the treatment of these gastrointestinal conditions.

Strengths and limitations of the systematic review and included studies

Strengths of the Systematic Review

The systematic review conducted by the research team demonstrated several notable strengths that contributed to the overall quality and reliability of the findings. Firstly, the authors' thorough search methodology was a key strength, as they performed a comprehensive search across multiple databases and registers. By ensuring that a significant number of relevant papers were included, this meticulous approach decreased the possibility of publication bias and improved the validity and resilience of the review's conclusions. Another strength of the review was the inclusion of a diverse range of study designs, incorporating both experimental RCTs and observational studies. This well-rounded perspective provided a more comprehensive understanding of the probiotics' effectiveness in treating adult patients with IBD and IBS.

Importantly, the review team prioritized the inclusion of high-quality RCTs, which are said to be the benchmark for determining how successful treatments are [36]. By focusing on these rigorous study designs, the review was able to reduce the risk of bias in the overall evidence synthesis, further strengthening the reliability of the findings.

The review team also demonstrated a commitment to a thorough assessment of the included studies' methodological quality, utilizing suitable tools such as the Cochrane RoB 2 tool [8] for RCTs and the NOS for observational studies [9]. This rigorous quality assessment process bolstered confidence in the review's findings by ensuring that the included evidence met robust standards of internal validity.

Finally, by incorporating studies from diverse sources and settings, the review was able to enhance the generalizability of its findings, enabling them to be used with a wider variety of people and contexts. This is a valuable strength, as it makes the review's results more pertinent and useful for clinical practice and decision-making.

Limitations of the Systematic Review

While the systematic review demonstrated numerous strengths, we recognize certain limitations that must be considered in assessing the results. One notable limitation was the lack of blinding in some of the included studies, where either the participants or the assessors were aware of the treatment allocation. This lack of blinding may have introduced bias into the results, potentially affecting the reliability of the study outcomes.

Additionally, certain studies included in the review had relatively low sample sizes, which could have impacted the generalizability of their findings and reduced the statistical power to detect significant effects. The review also identified that some studies had relatively brief intervals of follow-up periods, which may not have been sufficient to adequately capture the long-term impacts of the probiotic interventions.

Another limitation was the heterogeneity in the outcome measures utilized across the various studies. This variance posed a significant challenge in conducting a comprehensive meta-analysis. Additionally, we acknowledge that the scope of the search was limited to studies published exclusively in English, which may have inadvertently resulted in the exclusion of relevant evidence from non-English sources. This language restriction could have introduced a potential source of bias and reduced the comprehensiveness of the review.

Overall, while the review team used a thorough and rigorous process, the limitations that were found should be taken into account when interpreting the review's conclusions. Despite these drawbacks, the systematic review offers insightful information about the current level of evidence regarding the effectiveness of probiotic interventions in adult IBS and IBD patients, and it emphasizes the necessity of further investigation to fill in the gaps and address the limitations found in the body of existing research.

Future Research Directions

The comprehensive systematic review conducted by the research team, coupled with the insights gained from previous high-quality meta-analyses in this field, has highlighted several promising avenues for future research to further elucidate the efficacy of probiotic interventions in adult patients with IBS and IBD. One key area for future investigation is the need for well-designed, large-scale RCTs that employ rigorous blinding protocols to minimize the risk of bias. The review identified that some of the included studies lacked adequate participant or assessor blinding, which may have introduced potential sources of bias into the findings. By conducting larger, double-blind RCTs, researchers can generate more robust and reliable evidence on the true efficacy of probiotic treatments for IBS and IBD.

Additionally, future studies should aim to utilize more consistent and comprehensive outcome measures across research settings, addressing the heterogeneity in outcome assessment that was noted in the current review. The standardization of outcome measures would facilitate more robust meta-analyses, permitting a more thorough synthesis of the information that is already available and enabling clearer comparisons between different probiotic interventions.

Furthermore, the review highlighted the need for studies with extended follow-up periods to better understand the long-term impacts of probiotic therapies on disease management and symptom progression in IBS and IBD patients. Extending the duration of follow-up in future research will offer insightful information into the sustainability of probiotic-mediated effects and their potential to modify the natural course of these chronic gastrointestinal conditions.

## Conclusions

The effectiveness of probiotics in treating IBS and IBD varies significantly, as highlighted by this systematic review. The RCT results for IBS are mixed; while some studies demonstrate significant improvements in symptom scores, quality of life, and specific symptoms like abdominal pain and bloating with probiotic interventions, others show no statistically significant differences compared to placebo. In contrast, the evidence for probiotic use in IBD, particularly UC, is more promising, with several RCTs and observational studies reporting positive outcomes such as improved clinical results, reduced relapse rates, and better remission maintenance, especially with specific formulations like the De Simone combination. However, the effectiveness of probiotics for CD remains debatable, indicating a need for further research. Overall, this review underscores the necessity for well-designed, larger-scale RCTs with rigorous blinding, standardized outcome measures, and longer follow-up periods to clarify the optimal probiotic strains, dosages, and patient populations that may benefit from these interventions. Addressing the identified research gaps will enhance our understanding of probiotics in managing IBS and IBD.
